# Functional Integration of the Conserved Domains of Shoc2 Scaffold

**DOI:** 10.1371/journal.pone.0066067

**Published:** 2013-06-21

**Authors:** Myoungkun Jeoung, Lina Abdelmoti, Eun Ryoung Jang, Craig W. Vander Kooi, Emilia Galperin

**Affiliations:** 1 Department of Molecular and Cellular Biochemistry, University of Kentucky, Lexington, Kentucky, United States of America; 2 Department of Molecular and Cellular Biochemistry and Center for Structural Biology, University of Kentucky, Lexington, Kentucky, United States of America; Ohio State University, United States of America

## Abstract

Shoc2 is a positive regulator of signaling to extracellular signal-regulated protein kinases 1 and 2 (ERK1/2). Shoc2 is also proposed to interact with RAS and Raf-1 in order to accelerate ERK1/2 activity. To understand the mechanisms by which Shoc2 regulates ERK1/2 activation by the epidermal growth factor receptor (EGFR), we dissected the role of Shoc2 structural domains in binding to its signaling partners and its role in regulating ERK1/2 activity. Shoc2 is comprised of two main domains: the 21 leucine rich repeats (LRRs) core and the N-terminal non-LRR domain. We demonstrated that the N-terminal domain mediates Shoc2 binding to both M-Ras and Raf-1, while the C-terminal part of Shoc2 contains a late endosomal targeting motif. We found that M-Ras binding to Shoc2 is independent of its GTPase activity. While overexpression of Shoc2 did not change kinetics of ERK1/2 activity, both the N-terminal and the LRR-core domain were able to rescue ERK1/2 activity in cells depleted of Shoc2, suggesting that these Shoc2 domains are involved in modulating ERK1/2 activity.

## Introduction

Activation of mitogen-activated protein kinase/extracellular stimulus-regulated kinase 1 and 2 (MAPK/ERK1/2) initiates a complex network of signaling events that is regulated by scaffold proteins [1]–[3]. These proteins determine specificity of signaling outcomes by assembling unique signaling complexes at particular subcellular localizations and controlling the information transfer dynamics [4]–[6]. Scaffolds of the ERK1/2 signaling cascade tether and target components of the multi-protein signaling modules to various cellular locations (e.g. plasma membrane, endosomes, Golgi), thus ensuring accessibility of specific substrates [7]–[11]. Changes in the stoichiometric ratio of scaffold proteins and their binding partners may lead to titration of partner proteins into separate complexes, thus inhibiting their interaction [12].

Due to their function as multivalent adaptor proteins, scaffolds are often comprised of diverse structural and catalytic domains that indicate potential functions and even putative partners of the particular scaffold [13], [14]. An example of the functional complexity accomplished by the combination of multiple domains is Kinase Suppressor of Ras 1(KSR1) [15], [16]. KSR1 is a protein that contains five conserved domains: among them are a proline-rich sequence, a cysteine-rich domain that mediates interactions with membrane lipids, a serine/threonine-rich region that binds ERK/MAPK; and the putative kinase domain [8]. Other ERK1/2 pathway scaffold proteins may not carry catalytic motifs, but do have a number of protein interacting domains, including a STERILE α-MOTIF (SAM), a PDZ domain, proline-rich Src-homology-3 (SH3)-binding sites and a PH domain [8], [17]. The combination of domains composing the scaffold reflect the function of the scaffold and the types of plausible interactors [4].

Shoc2 is a critical modulator of the ERK1/2 pathway and was first identified in *C. elegans* (named SOC-2/SUR-8) [18]–[20]. Shoc2 forms a ternary complex with Ras and Raf-1 proteins [21], thereby positively regulating Ras-mediated signaling [19]. More recently, it was demonstrated that Shoc2 regulates ERK1/2 activity as a part of a holoenzyme comprised of Shoc2 and the catalytic subunit of protein phosphatase 1c (PP1c) [22]. Shoc2 was proposed to recruit PP1c to RAF-1 where PP1c dephosphorylates an inhibitory serine residue allowing for subsequent activation of RAF-1. Moreover, it was shown that Shoc2 modulates Ras-dependent Raf-1 activation in a Ca(2+)- and calmodulin-dependent manner [23], [24] in addition to reserving Ras-GTP for Raf-1, accelerating Ras-GTP binding to Raf-1, and enabling rapid temporal response to EGFR stimulation. Another study found that the S2G mutation of Shoc2 causes Noonan-like syndrome by promoting aberrant protein N-myristoylation and results in Shoc2 plasma membrane targeting [25]. A Shoc2 mouse knockout revealed that Shoc2 is essential for embryonic heart development [26]. We recently reported that Shoc2 translocates from the cytosol to late endosomes upon EGFR activation, while translocation of the S2G mutant of Shoc2 to late endosomes is impaired [27].

The basis for the Shoc2 function in accelerating ERK1/2 activity is unclear since it has only one recognized sequence – the leucine-rich repeats (LRR) domain and does not have a modular organization of other scaffold proteins. LRR proteins form a large family of intracellular, extracellular, and membrane-attached mostly eukaryotic proteins with cellular functions ranging from immune response and signal transduction to cell adhesion, RNA splicing and synapse development and functioning [28]–[32]. Despite their functional diversity, it is proposed that most LRR proteins participate in some form of protein-protein interactions and share a common, “solenoid”-like structure, with each LRR being a turn of the solenoid. Unique homogeneous structure of Shoc2 without obvious catalytic motifs or other interacting domains makes studies of Shoc2 particularly challenging. Therefore, to further our understanding of how Shoc2 regulates ERK1/2 signaling, we designed a structure-function study addressing the significance of particular Shoc2 regions for Shoc2 function and its cellular localization. We determined that an N-terminal domain, located prior to the LRR domain of Shoc2, contains an M- Ras-binding motif, while Shoc2 segment responsible for Shoc2 targeting to late endosomes is located at the C-terminus of Shoc2. We observed that M-Ras mediates interaction of Raf-1with Shoc2. Binding of Shoc2 with M-Ras is independent of the ERK1/2 pathway activation and M-Ras GTPase activity. Using a combination of shRNA and Shoc2 rescue approaches, we demonstrate that both, the N-terminal and the LRR domains are supporting the Shoc2 function in controlling ERK1/2 activity.

## Results

### Defining the LRRs in Shoc2

**Figure 1:**
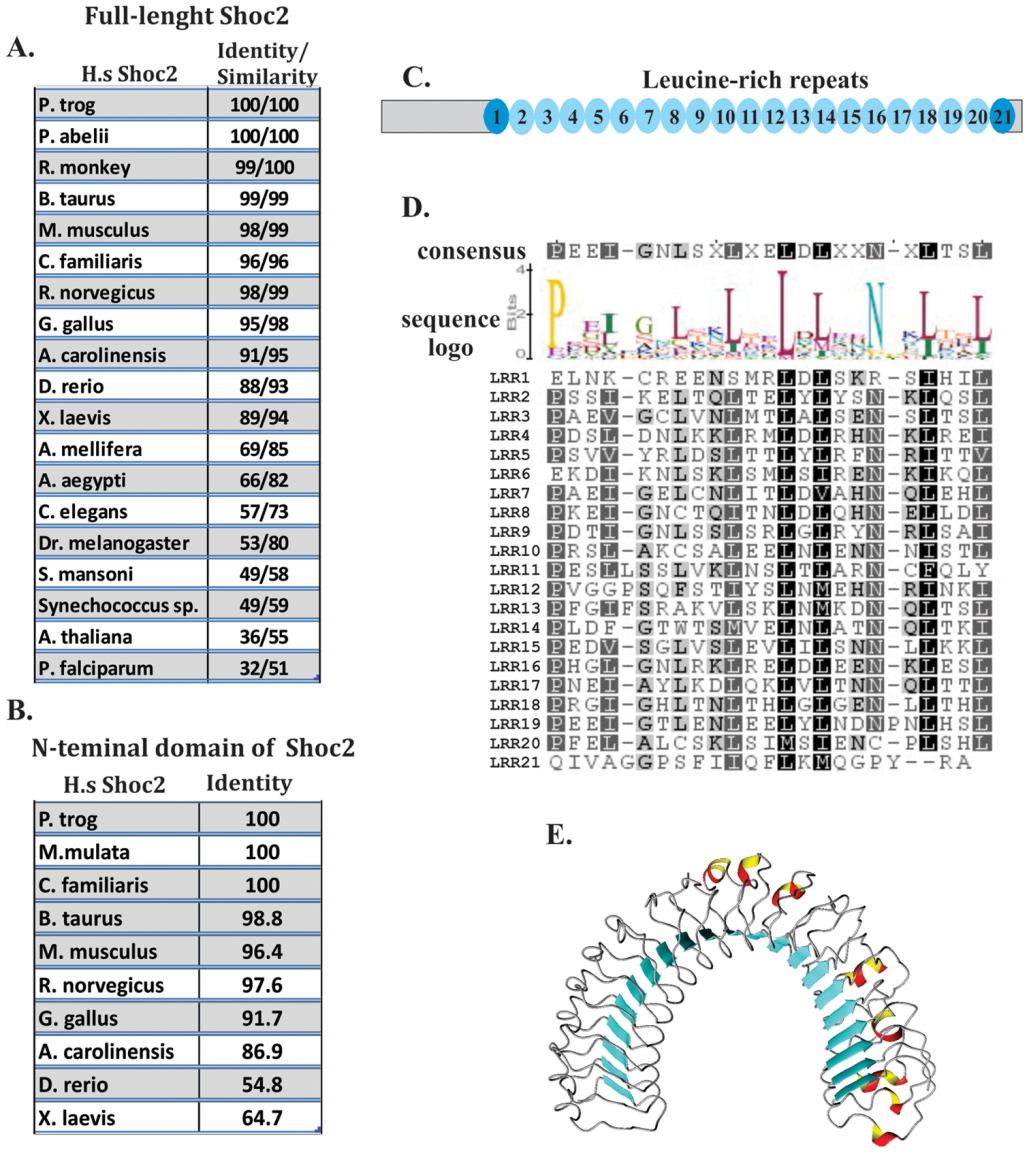
Defining the LRRs of Shoc2. ***A*** **,** Protein sequences from twenty two Shoc2 orthologues were utilized to generate an alignment of full-length Shoc2. The alignment was use to define the percent of similarity and identity of each Shoc2 orthologue to human Shoc2. ***B*** **,** Protein sequences from ten Shoc2 vertebrate orthologues were utilized to generate an alignment of the N-terminal domain. The alignment was use to define the percent of identity of each Shoc2 orthologue to human Shoc2. ***C***, Schematic representation of Shoc2 LRR and non-LRR regions. ***D,*** Multiple alignment of the individual twenty one LRRs of human Shoc2. The motif includes conserved sequence positions for the LRRs. Residue conservation color scheme: residues boxed in black are identical, and those boxed is dark and light grey are conserved substitutions. ***E***, Ribbon representation of the model structure of Shoc2. Three structure elements characterize the fold of this protein: tandemly repeating units – LRRs, the β sheets that occupy the concave face of the scaffold and a regular array of possible helixes that characterizes its convex face.

First, to determine the contribution of the individual structural components of Shoc2 to its function as a positive modulator of ERK1/2 activity, we defined the LRR boundaries. Although Shoc2 homologues in several organisms have been identified earlier [19], we have extended the sequence analysis using profile based sequence comparison to achieve a maximal resolution in sequence evaluation. Multiple Shoc2 orthologues in five classes of vertebrates (mammalia, aves, amphibia, reptilia and actinopterygii) were readily recognized by the National Center for Biotechnology Information's (NCBI) BLASTp analysis using human Shoc2 as the index sequence. In addition, we identified Shoc2 orthologues in several widely disparate group of organisms including cyanobacteria, plasmodium, parasitic trematode, nematode, plants (rice and arabidopsis), and insects (fruit fly, yellow fever mosquito, and honey bee) (Supporting Information 1). Although, most of the Shoc2 vertebrate orthologues were very consistent in size and contained approximately 580 amino acids, its other orthologues varied in size from 309 to 1009 amino acids. To ensure that we had identified Shoc2 orthologues and to determine their percent of similarity and identity to *H. sapiens* Shoc2, we used the Geneious^TM^ software package and generated pairwise alignments of 22 proteins. The similarities ranged from 51% for the plasmodium orthologue of Shoc2 to 100% for primate orthologues (Figure 1A). In addition to the LRR domain, we found that the N-terminal domain of Shoc2 was highly conserved in amino acid composition and length (85 amino acids) among mammalia, aves, reptilia and amphibia (e.g. 64.7% identity between human and frog) (Figure 1A and Figure S1).

Secondary structure prediction and multiple alignment analysis showed that the number of LRRs in Shoc2 is 21 (23 to 24 amino acids in length) in mammals (Figure 1B), amphibians, reptiles, actinopterygii and drosophila; 18 in aves; and variable numbers in plant orthologues. The repeats of all Shoc2 orthologues contain a highly conserved consensus, P*xx*I*xx*L*xx*L*xx*L*x*L*xx*N*x*L*xx*L, and encompass a consensus that is typically present in other LRR proteins, L*xx*L*x*L*xx*N*x*L (x being any amino acid) (Figure 1C). Notably, sequence conservation in the secondary structure (β-strands) was rather remarkable and aligned well with typical secondary structure of LRR proteins [33], [34], suggesting that these sequences may be involved in conserved interactive functions.

LRR structures are often flanked by N-terminal (LRRNT) and C-terminal (LRRCT) capping motifs that protect the hydrophobic core of the first and the last LRRs. This consensus sequence usually contains four cysteines in a CxnCxCxmC pattern, with *n* and *m* being variable numbers [35], [36]. We could not identify typical LRRNT or LRRCT motifs within Shoc2. However, we found that the N- and C-terminal LRRs (amino acids 92 to 115 and 558 to 580) showed lower similarity to the other “true” LRR sequences and probably serve capping functions, often observed in LRR domains and essential for stabilizing the fold of the protein. Indeed, the protein half-life of the Shoc2 truncated mutants lacking the N-terminal domain and the first LRR (aa116–582) is significantly shorter (∼2 hours) than that of the full-length Shoc2 (∼24 hours) (Figure S2B). Interestingly, with the exclusion of the N- and C-capping repeats, the percentage of identity in “true” LRR regions ranged from 54.8% for a parasitic trematode orthologue of Shoc2 to 100% for primate orthologues, which was significantly higher than the similarity/identity range for the full-length Shoc2 orthologues.

Utilizing homology and threading based modeling, a molecular model of the full LRR domain of Shoc2 was constructed (Figure 1D). The model clearly supported the 21 repeats in the Shoc2 LRR domain and not 18 LRRs, as was previously suggested [19]. This model of the LRR domain architecture was utilized in our subsequent engineering of Shoc2 truncation mutants. Taken together, these data indicated that the mammalian Shoc2 contains a conserved 21-repeat LRR domain. This data also suggested that the N-terminal domain may represent a distinct domain that is significant for Shoc2 function.

### Localization of Shoc2 truncated mutants

**Figure 2:**
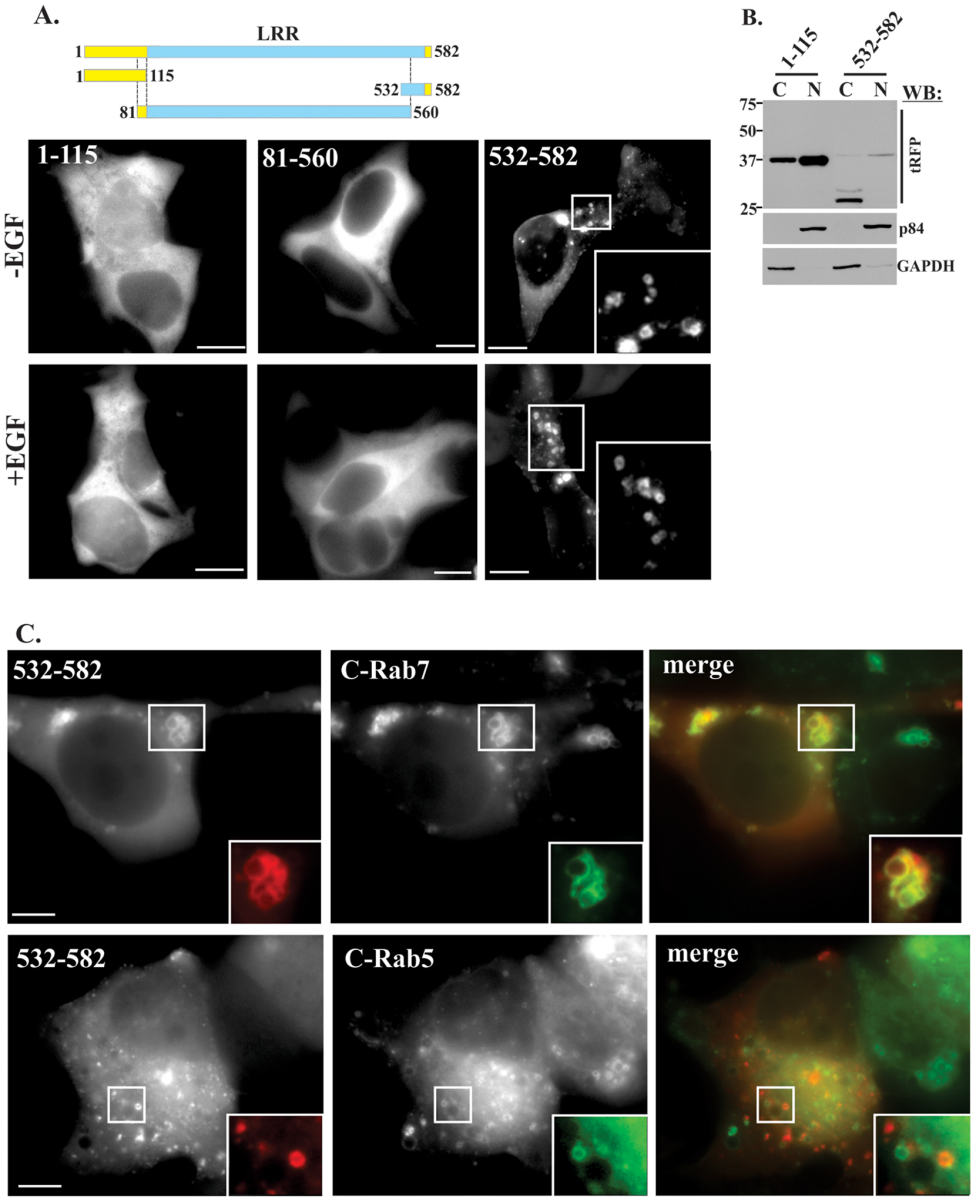
Localization of Shoc2 truncated mutants in Cos-LV1 cells. ***A***, Cos-LV1 cells were transfected with Shoc2-tRFP truncated fragments and imaged live before and after EGF treatment (0.2ng/ml, 15 min at 37°C). Shoc2-tRFP was located in the cytosol and nucleus. Scale bar, 10 µm. ***B*** **,** Cos-LV1 cells were transfected with full-length Shoc2-tRFP and its truncated fragments, and subjected to the cell fractionation. Cell lysates were immunoblotted (IB) with tRFP Abs to monitor expression of Shoc2, and p84 (nuclear fraction-N) Abs and GAPDH (cytosolic faction-C) to control for the purity of fractions. 30 µg of total lysate for each fraction was loaded for IB analysis. Results in each panel are representative of three independent experiments. ***C,*** Shoc2-tRFP mutant (532–582aa), CFP-Rab7 and CFP-Rab5 were transiently expressed in Cos-LV1 cells. Cells were serum-starved for 16h and then treated as in ***A***. Insets show high magnification images of the regions of the cell indicated by white rectangles, Scale bar, 10 µm.

To define the structural elements critical for Shoc2 function as a positive regulator of the ERK1/2 pathway and to determine which part of molecule confers the delivery of Shoc2 to endosomes, we prepared several truncated versions of monomeric tag red fluorescent protein (tRFP)-tagged Shoc2 starting with separating the LRR region from the non-LRR N-terminus (Figure 2A). Truncations were expressed in Cos-LV1 cells depleted of endogenous Shoc2 [27]. Analysis of the subcellular localization of Shoc2-tRFP truncated mutants using live-cell fluorescence microscopy demonstrated that the N-terminal domain (aa1-115) was found in cytosol and in the nucleus in Cos-LV1 cells before or after stimulation with EGF (Figure 2B). Cytosolic/nuclear localization of the N-terminal domain was confirmed by subcellular fractionation (Figure 2B). The LRR-domain of Shoc2-tRFP (aa81–560) was found mostly in cytosol. Surprisingly, the Shoc2 truncated mutant that spans the two C-terminal LRRs (aa532–582) when expressed in Cos-LV1 cells was found in vesicular structures before or after stimulation with of EGF. These vesicles appeared as fluorescent dots or as donut-like structures with occasionally visible limiting membrane (Figure 2B). The majority of the vesicles showed rapid lateral and directed movement over long distances, while others were relatively static (Movie S1). These vesicular structures localized to the Rab7-positive endosomes but did not co-localize with Rab5-positive endosomes in living cells (Figure 2C). The localization of this fragment to Rab7-positive endosomes was particularly easy to detect on a fused Rab7-positive compartment, presumably due to overexpression of CFP-Rab7. These data suggested that LRRs 20–21 at the C-terminus of Shoc2 contain sequences responsible for targeting of Shoc2 to Rab7 positive late endosomes, and other regions may structurally hinder these sequences.

### Identification of the binding regions for the immediate protein partners– M-Ras and RAF-1

To understand a role the Shoc2′s domains play in its function as a positive regulator of ERK1/2 signaling we mapped regions that are responsible for the direct binding to its immediate signaling partners Ras and Raf-1. Given that ablation of Shoc2 in mice led to embryonic lethality due to severe heart defects, we performed a yeast two-hybrid screen using a human ventricle and embryo heart library and full-length Shoc2 as bait. Twenty-tree positive clones encoding to M-Ras cDNA (aa1-199) and no other Ras isoforms (e.g. K-Ras, H-Ras or N-Ras) were identified during this screen. Thus, in the following experiments we have used an M-Ras isoform to define structural elements of Shoc2 that are essential for the M-Ras binding.

**Figure 3:**
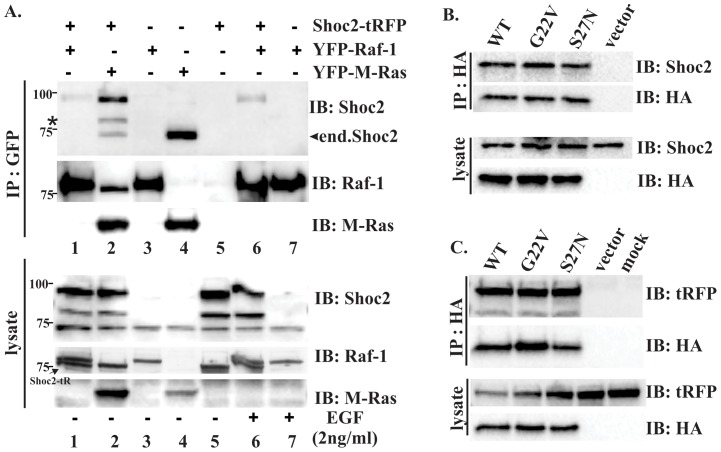
Shoc2 interaction with M-Ras and Raf-1. ***A,*** 293FT cells transiently co-transfected with expression vectors encoding full-length Shoc2-tRFP, YFP-Raf-1 or YFP-M-RAS. Twenty-four hours post-transfection, cells were starved for 16 hours, stimulated with EGF (2 ng/ml) for 15min and lysed. YFP-Raf-1 or YPF-M-Ras were immunoprecipitated (IP) using GFP antibody, and the immunoprecipitates were probed by immuno-blotting (IB) with Shoc2 and, subsequently, with GFP- and M-Ras antibodies to detect Raf-1 and Ras. Cell lysates were first immunoblotted with anti-Shoc2 antibodies to monitor Shoc2 expression, and then immunoblotted with anti-Raf-1 antibodies to monitor expression of YFP-Raf-1 and M-Ras antibody to monitor expression of YFP-M-Ras proteins. * denotes a proteolytic fragment of Shoc2-tRFP that is often detected by IB in cells expressing full-length Shoc2-tRFP. Shoc2-tRFP signal that is visible on the Raf-1 blot is indicated. ***B.*** 293FT cells transiently expressing HA-M-Ras and HA-M-Ras mutants (S27N and G22V). Thirty-six hours post-transfection, cells were lysed. M-Ras was immunoprecipitated using HA antibody, and the immunoprecipitates were probed by IB with Shoc2 and, subsequently, with HA-antibodies to detect Raf-1 and Ras. Cell lysates were immunoblotted with anti-HA antibody to monitor expression of corresponding M-Ras mutants used in panel IP or Shoc2 Abs to monitor expression of endogenous Shoc2. ***C***. Cos-SR cells transiently expressing HA-M-Ras and its mutants were subjected to IP as in ***B***. M-Ras was immunoprecipitated using HA antibody, and the immunoprecipitates were probed by IB with Shoc2 and, subsequently, with HA-antibodies to detect M-Ras. Cell lysates were immunoblotted with anti-HA antibody to monitor expression of corresponding M-Ras mutants used in panel IP or tRFP Abs to monitor expression of Shoc2-tRFP. Results in each panel are representative of three independent experiments.

In our initial experiments we tested the Shoc2 – M-Ras and -Raf-1 interaction using Shoc2 tagged with tRFP fluorescent protein (Shoc2-tRFP) and YFP-tagged M-Ras or Raf-1 co-expressed in 293FT cells (Figure 3A). YFP-Raf-1 immunoprecipitated Shoc2-tRFP (lane 1), but failed to precipitate endogenous Shoc2 (lane 3), possibly due to changes in the stoichiometric ratio of the components of the Shoc2 complex. Stimulating cells with 2ng/ml of EGF had a little effect on Shoc2-Raf-1 binding (lanes 6 and 7), suggesting that while Shoc2 is in complex with Raf-1 the activation of the ERK1/2 pathway does not lead to the recruitment of Raf-1 to Shoc2. Conversely, YFP-M-Ras was capable of efficiently precipitating both Shoc2-tRFP (lane 2) and endogenous Shoc2 (lane 4) even under the condition of serum starvation, though these results were somewhat in contrast to what was previously reported for the Shoc2 binding of M-Ras and Raf-1 [22]. To further explore this discrepancy, we performed co-transfection experiments to compare binding of GTP-bound and GDT-bound M-Ras proteins to Shoc2. Results in Figures 3B and C show that both mutants of HA-tagged M-Ras, GTP-bound (G22V) and GDP-bound (S27N) [37], maintained their capacity to precipitate Shoc2, suggesting that Shoc2-M-Ras binding is independent of GTPase activity of M-Ras.

**Figure 4:**
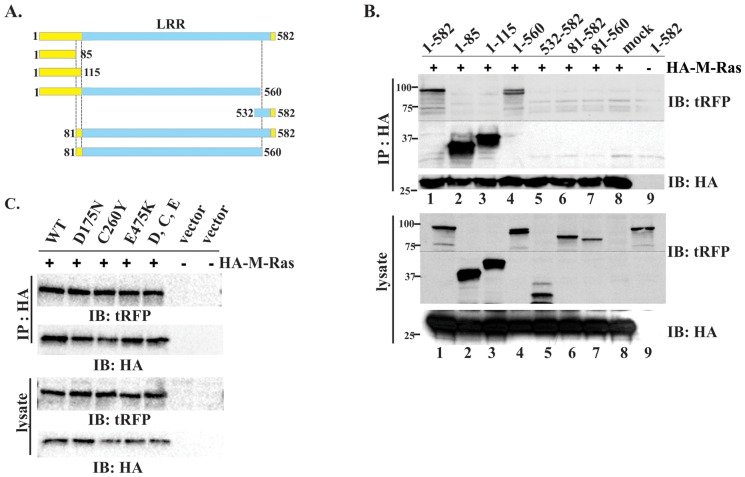
Shoc2 binding of M-Ras in 293FT cells. ***A*** **.** Schematic representation of the full-length and truncated Shoc2-tRFP constructs. ***B***, 293FT cells were transiently co-transfected with full-length or truncated tRFP-tagged Shoc2 and 3xHA-M-RAS. Thirty-six hours post-transfection, cells were lysed and lysates were subjected to immunoprecipitation (IP) with HA antibody. The IP fraction was analyzed by immuno-blotting (IB) with tRFP and, subsequently, with HA-antibodies to detect Shoc2 and Ras. Cell lysates were immunoblotted with anti-HA antibody to monitor expression of Ras proteins or tRFP Abs to monitor expression of full-length Shoc2 and corresponding truncated mutants used in panel IP. Dotted line shows area of the blot that was cropped to minimize occupied space. ***C***. 293FT cells were transiently co-transfected with expression vectors encoding full-length or mutated tRFP-tagged Shoc2 and 3xHA-M-RAS. Thirty-six hours post-transfection, cells were lysed. Lysates were subjected to IP with anti-HA antibody as described in ***B***. Results in each panel are representative of three independent experiments.

To define the M-Ras binding region more closely, we prepared a series of truncated mutants of Shoc2-tRFP (Figure 4A). All mutants represented in this study are stable and have a half-life similar to full-length Shoc2 (Figure S2B). 293FT cells were transiently co-transfected with HA-M-Ras and generated Shoc2-tRFP constructs and the capacity of HA-M-Ras to bind Shoc2-tRFP was tested (Figure 4B). Surprisingly, only the full-length Shoc2 and the mutants containing the N-terminal domain of Shoc2 co-precipitated with HA-M-Ras (lanes 1–4). Shoc2 fragments lacking the N-terminal domain failed to co-precipitate with HA-M-Ras (lanes 5–8). We observed that amounts of the N-terminal fragments in precipitates were higher than that of other Shoc2 mutants suggesting that the detected interaction of the N-terminal domain of Shoc2 with HA-M-Ras is not just due to the protein overexpression in cells. This also indicated that the N-terminal domain of Shoc2 is essential and sufficient for the association of Shoc2 with M-Ras.

The previous studies have identified loss of function mutations (D175N, C260Y, E457D and P510L) in *C. elegans* homologue of Shoc2, SUR8 [19]. Two of these point mutations (D175N and E457D) have been reported to disrupt M-Ras binding to Shoc2 [22]. As shown in Figure 4C, corresponding mutations in the Shoc2-tRFP sequence had no effect on the Shoc2-tRFP-HA-M-Ras binding, supporting our findings that the Shoc2 and M-Ras interaction is mediated by the N-terminal domain of Shoc2.

**Figure 5:**
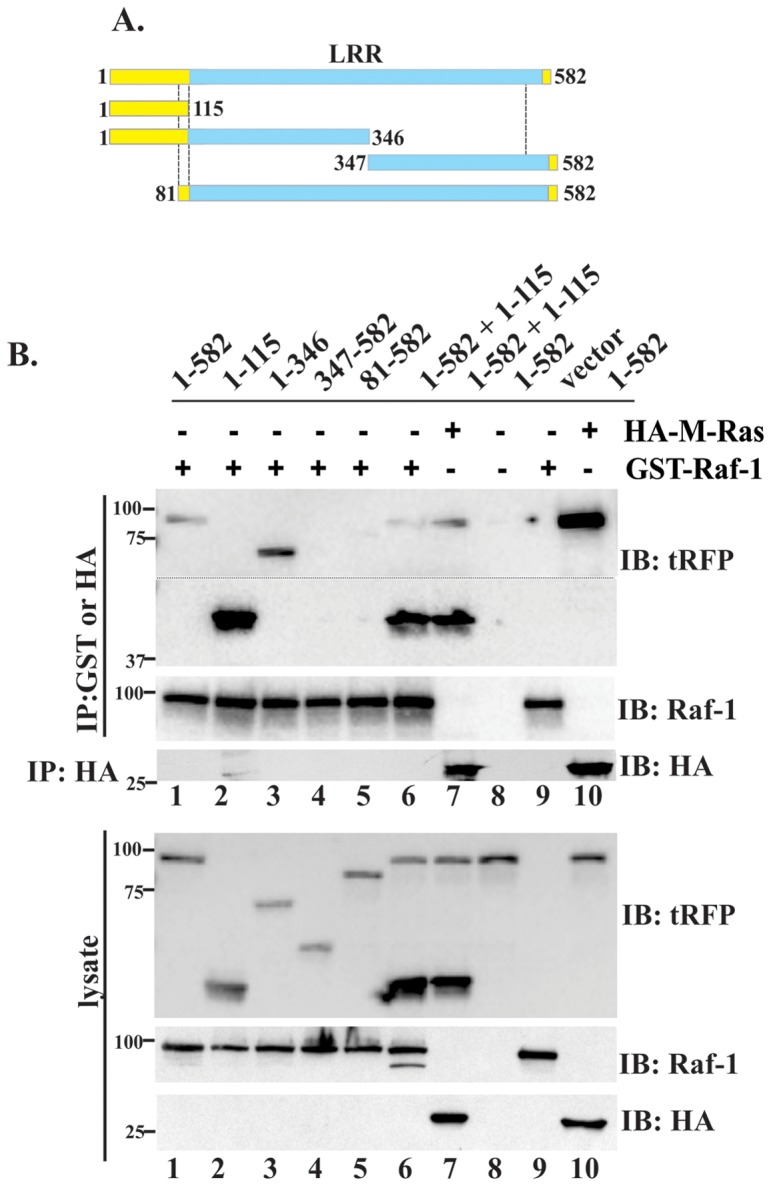
Shoc2 binding of Raf-1 in 293FT cells. ***A.*** Schematic representation of the full-length and truncated Shoc2-tRFP constructs. ***B*** *.* 293FT cells were transiently co-transfected with expression vectors encoding full-length or truncated tagRFP-tagged Shoc2 and YFP-Raf-1 or YFP-M-RAS. Thirty-six hours post-transfection, cells were lysed. GST-Raf-1 was precipitated with glutathione-coupled beads. HA-M-Ras was immuno-precipitated (IP) with HA antibody. The precipitated fraction was analyzed by immuno-blotting (IB) with tRFP and subsequently with Raf-1 and HA-antibodies to detect Raf-1 and Ras. Cell lysates were immunoblotted with Raf-1 antibodies to monitor expression of GST-Raf-1, HA antibodies to monitor expression of HA-M-Ras proteins, and tRFP antibodies to monitor expression of Shoc2 and its corresponding mutant used in panel IP. Results in each panel are representative of three independent experiments.

Given that Shoc2 was previously reported to interact with both Ras and Raf-1 proteins, we next ascertained the nature of Shoc2 and Raf-1 binding and determined a region that mediates Shoc2 association with Raf-1. To that end, 293FT cells were transiently transfected with the Shoc2-tRFP truncated mutants (Figure 5A and Figure S3A), GST-Raf-1, and HA-M-Ras and GST-Raf-1 was used to precipitate Shoc2 (Figure 5B). As expected, the interaction of the full length Shoc2-tRFP with Raf-1was much weaker (lane1) than it's interaction with HA-M-Ras (lane 10). Mutants containing the N-terminal domain of Shoc2 readily precipitated with GST-Raf-1 (lanes 2 and 3), while the Shoc2-tRFP truncations lacking the N-terminal domain failed to co-precipitate with GST-Raf-1(lanes 4 and 5). Moreover, GST-Raf-1 precipitated the N-terminal M-Ras binding domain (aa1-115, lane2) surprisingly well Figure S3B). Thus, we next tested whether the N-terminal domain of Shoc2 will compete for Shoc2 interaction with Raf-1 or M-Ras. Cells expressing GST-Raf-1 (lane 6) or HA-M-Ras (lane 7) were transfected with both Shoc2-tRFP and the N-terminal domain of Shoc2 (aa1-115). We observed reduced binding of the full-length Shoc2-tRFP to HA-M-Ras (lane 10 vs. lane 7) and GST-Raf-1 (lane 1 vs. lane 6). These data suggested that the N-terminal domain of Shoc2 sequestered M-Ras in cells and implied that the interaction of Shoc2 with Raf-1 is mediated by M-Ras, presumably through its Raf-1binding domain.

### Functional status of Shoc2 truncated mutants

**Figure 6:**
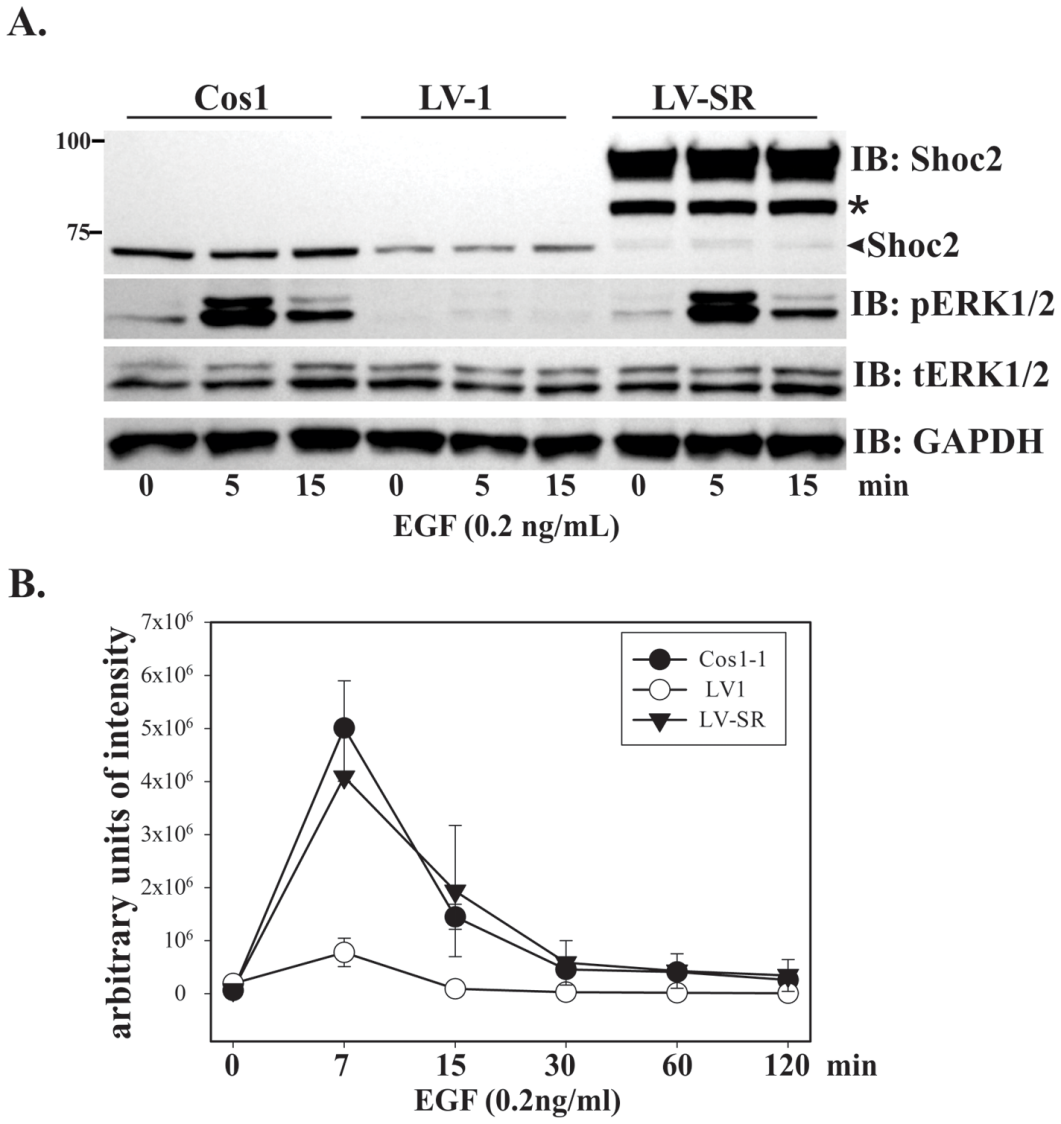
Overexpression of Shoc2 does not affect ERK1/2 activation by EGF in Cos1 cells. ***A,*** Parental Cos1 and Cos1 cells stably expressing Shoc2-shRNA (Cos-LV1) or Shoc2-shRNA and Shoc2-tRFP (LV-SR) were serum-starved up to 12 hours and treated with 0.2ng/ml EGF for indicated times at 370C. The lysates were probed for Shoc2, activated ERK1/2 (pERK1/2), total ERK1/2 (ERK1/2) and GAPDH (loading control). Results in each panel are representative of three independent experiments. * denotes a proteolytic fragment of Shoc2-tRFP that is detected by immuno-blotting (IB) in cells expressing full-length Shoc2-tRFP. ***B*** *,* Multiple blots from the experiments exemplified in ***A*** were analyzed.

To further analyze the role of the Shoc2′s domains in regulating ERK1/2 signaling initiated by EGFR, we generated Cos1 cells (LV-SR) that have endogenous Shoc2 replaced with Shoc2-tRFP. In these cells endogenous Shoc2 was stably silenced by shRNA and replaced by ectopically expressed Shoc2-tRFP. In Shoc2-tRFP 6 “silent” mutations were introduced to generate it to be resistance to shRNA without changing its amino acid sequence [27]. The control cells expressing either non-targeting shRNA (LV-NT) or Shoc2 specific shRNA only (LV1) have been developed and reported previously, and have been used in the following experiments of this study. The effect of Shoc2 knockdown on the ERK1/2 pathway activation was most evident when the cells were stimulated with low, physiological (0.2 ng/ml) concentration of EGF [27], thus in following experiments cells were stimulated with the corresponding concentration of EGF. LV-SR cells were next utilized to assess whether overexpression of Shoc2 affects the amplitude or the duration of ERK1/2 activity. In order to prevent clonal variations due to the different sites of viral genome incorporation in our experiments we used a pool population of LV-SR cells, constitutively expressing Shoc2 shRNA and Shoc2-tRFP. Data presented in Figure 6 show that Cos1 and LV-SR cells had comparable amplitude and duration of ERK1/2 activation when stimulated with low concentration of EGF (0.2 ng/ml) for a maximum period of 120 min. These data also demonstrated that Shoc2-tRFP is capable of fully rescuing EGF-induced ERK1/2 phosphorylation when expressed constitutively, and an increase in Shoc2-tRFP levels in cells does not affect the kinetics of ERK1/2 signals.

**Figure 7:**
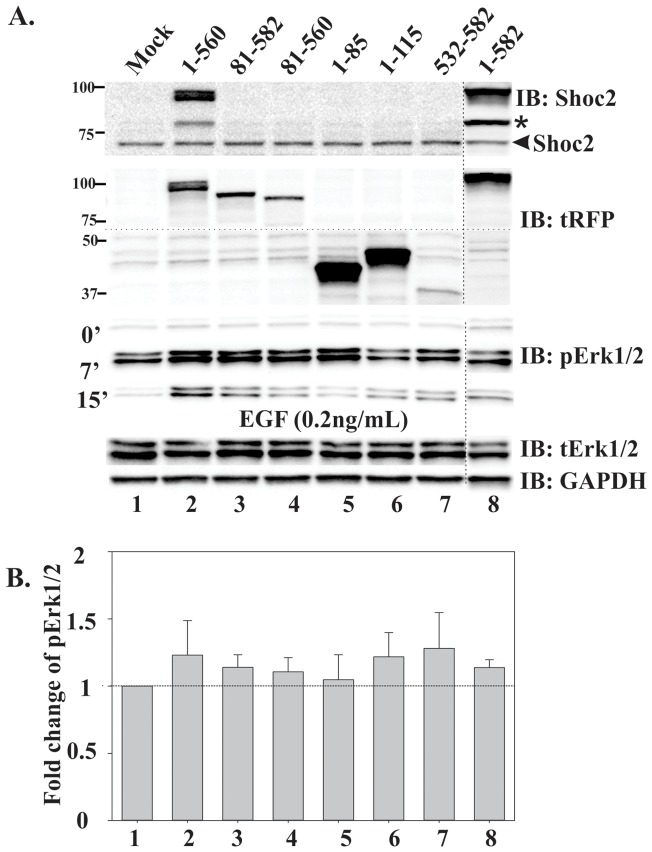
Wild-type Shoc2 and Shoc2 truncated mutants do not have a dominant-interfering effect in Cos-NT cells. ***A***, Cos1 cells were transiently transfected with full-length Shoc2-tRFP or Shoc2-tRFP truncated mutants. Cells were serum-starved for up to 12 hours and treated with 0.2 ng/ml EGF for indicated times at 37°C. The lysates were probed by immuno-blotting (IB) for Shoc2, tRFP, activated ERK1/2 (pERK1/2), total ERK1/2 (tERK1/2) and GAPDH (loading control). Dotted line shows area of the blot that was cropped to minimize occupied space. * denotes a proteolytic fragment of Shoc2-tRFP that is detected by immuno-blotting (IB) in cells expressing full-length Shoc2-tRFP. ***B***, Multiple blots from the experiments exemplified in ***A*** were analyzed. Bars represent the mean values (±S.E., *n* = 4) of phosphorylated ERK1/2 activity normalized to total ERK1/2 in arbitrary units (pERK1/2/ERK), P = 0.938 (one-way ANOVA test was used to determine differences in phosphorylated ERK1/2 activity).

Previous studies showed that overexpression of scaffold proteins followed by changes in stoichiometry of the complex components may lead to an increase or inhibition of the ERK1/2 pathway activity [10]. As the Shoc2 signaling partners M-Ras and Raf-1 interact with the N-terminal domain of Shoc2, we reasoned that the mechanism underlying the ability of Shoc2 to accelerate activation of the ERK1/2 pathway may be supported by this region of Shoc2. Thus, we next tested whether expression of the Shoc2-tRFP truncated mutants will affect activation of the ERK1/2 pathway in the presence of endogenous Shoc2 (Figure 7A and Figure S4). Shoc2 antibodies recognize an epitope on the N-terminus of Shoc2, thus to simultaneously detect expression of endogenous Shoc2 and all the Shoc2-tRFP truncated mutants utilized in this study we first probed our western blots with Shoc2 and then with tRFP antibody. All Shoc2-tRFP constructs had 6 “silent” mutations introduced to render them resistant to shRNA without changing the amino acid sequence, as reported earlier [27]. Surprisingly, expression of Shoc2-tRFP truncated mutants in Cos1 cells stimulated with low (0.2 ng/ml) concentration of EGF had no significant effect on ERK1/2 activation (Figure 7B). At the expression levels that were maximally achievable for the reported constructs in Cos1 cells, only a minimal increase in ERK1/2 phosphorylation (by factor of 0.5) was observed (Figure 7B). Expression of the N-terminal domain of Shoc2 (lane 6) and the truncated mutant that contains a late-endosomal targeting region (lane 7) also resulted in only a moderate increase in ERK1/2 phosphorylation.

**Figure 8:**
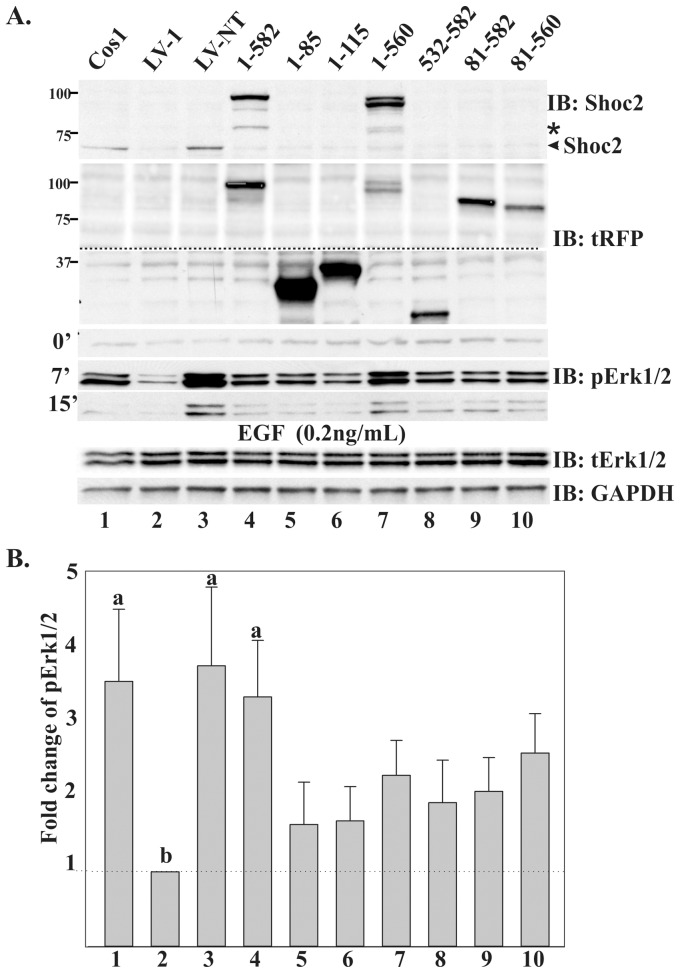
Wild-type Shoc2 and Shoc2 truncated mutants rescues Shoc2 knockdown. ***A***, Cos-LV1 cells were transiently transfected with full-length Shoc2-tRFP or Shoc2-tRFP truncated mutants. Cells were serum-starved for up to 12 hours and treated with 0.2ng/ml EGF for indicated times at 37°C. The lysates were probed by immuno-blotting (IB) for Shoc2, tRFP, activated ERK1/2 (pERK1/2), total ERK1/2 (tERK1/2) and GAPDH (loading control). Dotted line shows area of the blot that was cropped to minimize occupied space. * denotes a proteolytic fragment of Shoc2-tRFP that is detected by IB in cells expressing full-length Shoc2-tRFP. ***B***, Multiple blots from the experiments exemplified in ***A*** were analyzed. Bars represent the mean values (±S.E., *n* = 3) of phosphorylated ERK1/2 activity normalized to total ERK1/2 in arbitrary units (pERK/ERK), a vs. b, P<0.05 (one-way ANOVA followed by a post-hoc Student Newman-Keuls test was used to determine differences in phosphorylated ERK1/2 activity).

To test the capacity of Shoc2′s domains to rescue the ERK1/2 pathway activation, different Shoc2-tRFP truncated mutants were expressed in Cos-LV1 cells stimulated with 0.2 ng/ml of EGF (Figure 8A).Transient expression of the full-length Shoc2-tRFP in Cos-LV1 cells fully rescued ERK1/2 activation (by a factor of 3.5 to 4.5) (lane 4). The ERK1/2 phosphorylation signal in cells transiently expressing Shoc2-tRFP was slightly lower than in Cos1 or LV-NT cells, presumably, due to the fact that not all Cos-LV1 cells expressed Shoc2-tRFP. Expression of the N-terminal domain of Shoc2 in Cos-LV1 cells led to a partial recovery of ERK1/2 phosphorylation (by a factor of 1.5 to 2) (lanes 5 and 6). Unexpectedly, expression of other Shoc2 truncated mutants, including the truncations containing the endosomal targeting signal (lane 8), also restored ERK1/2 activity above the basal level of EGF-induced ERK1/2 activity in Shoc2-depleted cells (Figure 8B). These data indicate that multiple Shoc2 domains, including its N-terminus, LRR-core and endosomal targeting domain play a role in Shoc2 function as a positive modulator of ERK1/2 signaling.

In summary, our data demonstrate that, in addition to its LRR-core, the Shoc2′s N-terminal domain and its C-terminal endosome targeting region are the part of the mechanism that regulates ERK1/2 pathway activity induced by EGFR activation.

## Discussion

The ERK1/2 pathway scaffold protein Shoc2 belongs to a family of the LRR proteins that shares a common structural framework with repeats arranged to form a continuous super-helix [38]. This structure appears to be the principal reason that most of the LRR proteins are involved in protein-protein interactions [34], [39]. We explored and analyzed Shoc2 structural properties to elucidate the mechanisms underlying Shoc2′s function as a modulator of ERK1/2 signaling and Shoc2 distribution in cells.

As bioinformatic analysis demonstrates, Shoc2 is broadly distributed and highly conserved not only in vertebrates but also in many disparate taxa, with strikingly high levels of sequence similarity in its LRR domain (Figure 1A). Additionally, we found that the non-LRR N-terminal domain of Shoc2 is highly conserved in vertebrates, suggesting that the N-terminal domain represents a previously overlooked functionally important domain. These findings also indicate that both regions of Shoc2 (N-terminus and LRR core) retained their function during the course of evolution. Sequence differences in the N-terminus of Shoc2 between vertebrate and invertebrate orthologues may reflect diversion in function and/or variation in binding partners with the possibility of Shoc2 orthologues supporting distinct but related functions.

Molecular modeling and sequence analysis showed that the Shoc2 solenoid is built of 21 LRRs with each LRR containing the amino acid sequence L*xx*L*x*L*xx*N/C*x*L (*x* being any amino acid). The first and the last of the 21 LRRs are likely acting as capping repeats and stabilize the Shoc2 fold. This conclusion is also consistent with earlier demonstrations that LRRs of other proteins often have a significant degree of structural complementarity with their immediate neighboring repeats or capping structures covering them, and engineering truncated LRR domains with poor complementarity destabilizes them [28], [40].

In an effort to identify domains responsible for the spatial distribution of Shoc2, we found that the N-terminal truncated mutant of Shoc2 is localized in the cell nuclei regardless of the activation status of the ERK1/2 pathway (Figure 2). Further studies will explore whether this nuclear localization of Shoc2 is the result of Shoc2 overexpression, or a part of Shoc2′s trafficking route controlled by the mechanisms of nuclear import/export. We also found that the C-terminal fragment of Shoc2 (aa532–582), when isolated from the rest of the solenoid and tagged with tRFP, was targeted to the surface of late endosomes. It is tempting to speculate that the last two LRRs contain a novel recognition motif responsible for the Shoc2 delivery to endosomes. Since Shoc2 lacks any trans-membrane segments, this motif will likely associate with a protein and/or lipids at the periphery of the late endosome membrane. We previously demonstrated that full-length Shoc2 is delivered to late endosomes upon activation of the ERK1/2 pathway and suggested that the mechanism that governs targeting of Shoc2 to late endosomes requires greater temporal control. Thus, the newly identified endosome-binding interface of Shoc2 could be a part of such a mechanism directing dynamic changes in Shoc2 distribution. Future studies will unravel mechanisms and sequences/motif that are responsible for Shoc2 targeting to late endosomes.

Interaction of Shoc2 with Raf-1 and different members of Ras family have been reported in several conflicting studies [19], [20], [22]. Our yeast two-hybrid screen of human heart library with Shoc2 as a bait had recognized isolates of only one Ras isoform, M-Ras. Given that M-Ras is highly expressed in heart [37] and that Shoc2 knock-out mice exhibit major heart defects [26], it is possible that Shoc2-Ras complexes are tissue specific and M-Ras is a preferable Shoc2 partner in heart tissues. To our surprise, the immunoprecipitation experiments showed that the N-terminal domain of Shoc2 provides a binding surface to M-Ras (Figure 3 and 4). Although, previous reports by Li, *et al.* (2000) and Sieburth, *et al.* (1998) showed that deletion of the C-terminal 53 amino acids or even several C-terminal LRRs of the *C. elegans* orthologue of Shoc2 eliminated LET-60 Ras binding, we have not found the C-terminal region of human Shoc2 to be necessary for the binding of M-Ras (Figure 3). Moreover, binding of M-Ras to Shoc2 was independent of ERK1/2 pathway activation and the GTPase activity of M-Ras. Therefore, it is possible that in addition to having a preferred Ras partner in specific tissues, Shoc2 also uses discrete mechanisms to bind specifically different members of the Ras family (e.g. H-Ras, K-Ras, N-Ras), and further studies will define molecular determinants dictating this specificity. Our results also provide evidence that M-Ras mediates Raf-1 proximity with Shoc2 (Figure 3), which agrees with studies by Rodriguez-Viciana, *et al.* (2006) showing that Raf-1 does not interact directly with Shoc2.

Based on the results that the LRR domain is not engaged in M-Ras-Raf-1- binding, we suggest that the LRR domain is available to bind other partners of this scaffold complex or, perhaps, available to form stable dimers. It also indicates that mechanisms of Shoc2 function are more complex than a simple tethering of the Ras and Raf-1. These data coincide with a Shoc2 accelerator model proposed in the study by Matsunaga-Udagawa, R. *et al.* in which Shoc2 accelerated both the association and the dissociation of the Ras-RAF-1 interaction [23].

Another important finding was that in cells activated with the low, physiological concentrations of EGF overexpression of Shoc2-tRFP had no effect on activation of ERK1/2 in the presence of endogenous Shoc2 (Figures 7 and 8), though it was in contrast to what was observed in other studies when EGFR was activated using high EGF concentrations [25]. Outcomes of our experiments are reminiscent to what was observed in overexpression studies of other ERK1/2 scaffold proteins, KSR1and MP1/p14. Full-length KSR1 when expressed at high levels blocked ERK1/2 activation and the KSR-mediated R7 photoreceptor formation [41]–[44]. Overexpressed p14, a partner in the MP1/p14 scaffolding complex, did not have an effect on ERK1/2 signaling and could only significantly stimulate ERK1 activity *in vitro*, when stoichiometry of signaling complexes was not disrupted [45], [46]. MP1 expressed at various concentrations affected assembly of the MP1-MEK1-ERK1 signaling modules and ERK1/2 activity by either inhibiting or stimulating ERK1/2 signaling, again, relative to the stoichiometry of the complex components [47], [48]. Notably, our recovery studies revealed a role for both, the N-terminal and the LRR Shoc2 domains, in regulating EGFR-mediated activation of the ERK1/2 pathway. The N-terminal domain supports Ras-Raf-1 complex assembly, thus it was expected that this domain would rescue ERK1/2 activity. On the other hand, we did not expect to find that expression of other truncated mutants would accelerate ERK1/2 activity in Cos-LV1 cells (Figure 8). Furthermore, we did not expect to find that the rescue effects of the Shoc2 isolated domain on ERK1/2 activation did not vary with the levels of expressed protein. An attractive explanation for these observations would be that the Shoc2 domains combine two separate functionalities that exert both positive and negative effects on ERK1/2 signaling. When taken out of context of the full-length molecule, the Shoc2 N-terminal domain assembles Ras-Raf-1 complexes and rescues ERK1/2 activity. As for the LRR core, its expression will dilute other Shoc2 binding partners that negatively affect ERK1/2 activity. This model is consistent with that proposed by Matsuda and co-workers that implies a negative feed-back loop involving Shoc2 [24]. One possible partner is PP1c, as Shoc2 is a part of the PP1c holoenzyme [22]. However titration of PP1c alone cannot account for such an increase in ERK1/2 phosphorylation observed in rescue experiments.

Therefore, we propose that the function of Shoc2 goes beyond its role in tethering of Ras-Raf-1 and enforcing their physical proximity, but Shoc2′s function extends to the coordinating and integrating positive and negative ERK1/2 feedback loops. The LRR domain is the one that is most likely to govern the negative feedback loop. Further biochemical studies will assess the precise architecture of the complex formed by Shoc2 through LRR, determine the mechanisms coupling both feedback loops, and define the mechanisms governing assembly of the Shoc2 scaffold complexes.

## Materials and Methods

### Reagents and Antibodies

EGF was obtained from BD Bioscience (Bedford, MA). Antibodies to EGFR, RAF-1, ERK1/2, phospho-ERK1/2, M-Ras, and GAPDH were from Cell Signaling Technology; Shoc2 and GFP antibodies were from Abcam (USA); HA antibodies were from SydLabs; tRFP antibodies from Invitrogen, p84 antibodies were from GeneTex (Irvine, CA). Pfu polymerase was purchased from Stratagene (La Jolla, CA).

### Expression plasmids

Shoc2-tRFP, CFP-tagged Rab7 and Rab5 plasmids were described previously [49], [50]. The plasmid carrying YFP-Raf-1 was a gift from Dr. Sorkin (University of Pittsburgh). Truncated Shoc2-tRFP tagged mutants were generated as described for the full-length Shoc2 [27]. 3xHA-M-RAS plasmids were purchased from Missouri S&T cDNA Resource Center (www.cdna.org). To generate a plasmid stably expressing Shoc2 specific shRNA and Shoc2-tRFP, the pLVTHM-Shoc2 construct and Shoc2-tRFP construct resistant to shRNA knockdown were used (both were described previously [27]. Shoc2-tRFP cDNA resistant to shRNA knockdown containing *PmeI* and *NdeI* restriction sites was synthesized. cDNA was ligated into pLVTHM-Shoc2 using *PmeI* and *NdeI* restriction sites. The pLVTHM-Shoc2-Shoc2-tRFP construct was verified by dideoxynucleotide sequencing.

### Yeast two-hybrid (Y2H) screening

The full-length human Shoc2 was cloned into the lexA vector pB27 as an N-LexA-Shoc2-C fusion and screened against a human embryo ventricle and heart prey cDNA library. Y2H screens were performed by Hybrigenics SA (http://www.hybrigenics-services.com), Paris, France.

### Sequence data and analysis

The reference for all complete Shoc2 sequences from vertebrate and invertebrate species available on April 2012 were collected from the NCBI Entrez Nucleotide database (http://www.ncbi.nlm.nih.gov) using the Blastn and Blastp search algorithm (Supporting Information S1) (Altschul *et al.*, 1997). In our searches of databases for Shoc2 orthologues, we used the criteria that Shoc2 orthologues must have a maximum percentile of sequence similarity, a maximum number of LRRs and contain a non-LRR amino-terminal region. Orthologues of each member of the Shoc2 family were confirmed by a maximum likelihood tree using the Geneious 5.7.5 software. Multiple alignments of the amino acid sequences for the full lengths of each member were performed using the Geneious 5.7.5 software, and then aligned sequences were partitioned into LRR sequences based on LRRs consensus sequences identified prior [31]. Alignments of the LRRs were also checked by eye.

### Molecular Modeling

A model of the Shoc2 LRR domain encompassing residues 101–560 was constructed. The N-terminal repeats (residues 101–246) were modeled using A29 as a template (PDB = 2O6Q) [51], the middle repeats (residues 247–517) were modeled using BRI1 as a template (PDB = 3RGZ) [52], and the C-terminal repeats (residues 518–578) were modeled using TLR4 as a template (PDB = 2Z64) [51]. The N- and middle repeats were generated using the SWISS-MODEL automated prediction server [53] and C-terminal repeats using the Phyer2 protein threading server [54]. A well-packed representative model containing all repeats arranged in the expected LRR topology was constructed using least squares fit [55] of overlapping regions. Figures were prepared using MOLMOL [56].

### Cell Culture and DNA Transfections

293FT cells (Invitrogen), Cos-1(ATCC), and stable cell lines (Cos-LV1, LV-NT, LV-SR) (derivative of Cos-1 cells) were grown in Dulbecco Modified Eagle's Medium (DMEM) containing 10% fetal bovine serum (FBS) supplemented with Sodium Pyruvate, MEM-NEAA, Penicillin, Streptomycin, and L-Glutamate (Invitrogen). The transfections of DNA constructs were performed using PEI (Neo Transduction Laboratories, Lexington, KY) or *Trans*IT^®^ (Mirus Bio LLC) reagents. Expression of tagRFP-fused proteins was confirmed by Western blotting as described below.

### Immunoprecipitation and Western blot analysis

The Cos1 and 293FT cells grown in 35-mm dishes were placed on ice and washed with CMF-PBS, and the proteins were solubilized in 20 mM HEPES (Sigma) pH 7.6, 10 mM NaCl, 1.5mM MgCl_2_, 1 mM EDTA (Sigma), 1 mM EGTA (Sigma), 0.5 mM PMSF (Sigma), 10 µg/ml of leupeptin (Roche), 10 µg/ml of aprotinin (Roche), 5 µg/ml of pepstatin A (Sigma), 50 mM β-glycerophosphate (Sigma) lysis buffer for 15 min at 4°C [57]. Lysates were then centrifuged at 2,500×*g* for 15 min to remove the insoluble material. Lysates were incubated with appropriate antibodies for 2 h, and the immuno-complexes were precipitated using Protein A or G Sepharose. Immunoprecipitates and aliquots of cell lysates were denatured in the sample buffer at 95°C, resolved by electrophoresis, and probed by Western blotting with various antibodies followed by the chemiluminescence detection. Western blotting was done as described previously [58]. Glutathione-S-transferase (GST) pull-down was done similarly to the immunoprecipitation above, using glutathione-coupled beads for purification of the immunocomplex. Several x-ray films were analyzed to determine the linear range of the chemiluminescence signals, and the quantifications were performed using the densitometry analysis mode of the Image Lab software (Bio-Rad, Inc). In all experiments, statistical analyses were performed with one-way ANOVA followed by a post-hoc Student Newman-Keuls test or with a nonparametric Kruskal-Wallis test when required. These analyses were performed using SigmaStat3.5 (Systat Software, Inc. Point Richmond, CA).

### Fluorescence imaging of living cells

The cells were plated 24 hours before the experiment onto 35-mm glass-bottom dishes and kept in serum free and phenol red free medium containing 0.2% BSA for 16–20 hours. Cells grown on glass-bottom dishes were either treated or not treated with 0.2 ng/ml of EGF. All images were acquired using a Mariannas Imaging system consisting of a Zeiss inverted microscope equipped with a cooled CCD CoolSnap HQ (Roper, CA), dual filter wheels and a Xenon 175 W light source, all controlled by SlideBook 5.32 software (Intelligent Imaging Innovations, Denver, CO). The detection of tagRFP fluorescence was performed using a TRIC channel and CFP using a CFP channel. Images were acquired using 2×2 binning mode. Image analysis was performed using the SlideBook 5.32 software.

### Nuclear–cytoplasmic fractionation

Cos-LV1 cells were transfected with plasmids expressing different Shoc2 truncated fragments. After 48 hours of transfection, nuclear and cytosolic extracts were prepared using the NE-PER Nuclear and Cytoplasmic Extraction Reagents kit (Thermo Fisher Scientific) according to the manufacturer's protocol. Briefly, cells were washed once with cold PBS. Cells were collected by centrifugation at 500×*g* for 5 min. The pellet was resuspended in 200 µl of CER I (cytoplasmic extraction reagent I) and incubated on ice for 15 min. CER II (cytoplasmic extraction reagent II) was added and samples were vortexed for 5 seconds. Nuclei were collected by centrifugation at 10,000×*g* for 15 min at 4°C, and the supernatant was transferred to a pre-chilled tube for cytosolic fraction. The nuclei were then washed with cold PBS, centrifuged and resuspended in 100 µl NER (nuclear extraction reagent), incubated on ice for 15 min, and then centrifuged at 10,000×*g* for 10 min. Protein concentration was determined using Bio-Rad Protein Assay reagent. P84 was used as a nuclear and GAPDH was used as a cytosolic fraction control.

## Supporting Information

Supporting Information S1 (docx)

Figure S1 **Multiple alignment of the Shoc2 N-terminal domain.** An alignment of the N-terminal domains is to highlight the conservation of amino residues in vertebrate spices. The intensity of the background reflects the % of conservation of a given position within the 10 sequences (light grey>60%, dark grey>80%, black, full identity).
(PDF)

Figure S2 **Full-length Shoc2-tRFP and Shoc2-tRFP truncated mutants have differential protein half-life.** ***A*** *,* Schematic representation of the full-length and truncated Shoc2-tRFP constructs. ***B,*** Cos1 cells were transiently transfected with full-length Shoc2-tRFP or Shoc2-tRFP mutants. Thirty-six hours post-transfection cells were treated with 30 µM Cycloheximide for indicated times at 37°C. The lysates were probed by immuno-blotting (IB) for tRFP, Cyclin D and GAPDH (loading control).
(PDF)

Figure S3 **Shoc2 binding of Raf-1 in 293FT cells.** ***A,*** Schematic representation of the full-length and truncated Shoc2-tRFP constructs. ***B,*** 293FT cells were transiently co-transfected with expression vectors encoding full-length or truncated tagRFP-tagged Shoc2 and GST-Raf-1. Thirty-six hours post-transfection, cells were lysed. GST-Raf-1 was precipitated with glutathione-coupled beads. The precipitated fraction was analyzed by immuno-blotting (IB) with tRFP and subsequently with Raf-1 to detect Raf-1. Cell lysates were immunoblotted with Raf-1 antibodies to monitor expression of GST-Raf-1, and tRFP antibodies to monitor expression of Shoc2 and its corresponding mutant used in panel IP.
(PDF)

Figure S4 **Activation of the ERK1/2 pathway in Cos1, Cos-LV1 and Cos-SR cells.** Cos1, Cos-LV1, LV-SR, and Cos-1 cells transiently transfected with tRFP for 36 h were treated with EGF (0.2 ng/mL) for indicated times at 37°C. The lysates were probed by immuno-blotting (IB) for pErk1/2, Shoc2, tRFP, and total Erk1/2 (loading control).
(PDF)

Movie S1 **Cos-LV1 cells were transiently transfected with Shoc2-tRFP truncated mutants (aa532–582).** Time-lapse images were acquired every 5 sec during 3 min at room temperature.
(MP4)
