# Supplementary material for: Functional Integration of the Conserved Domains of Shoc2 Scaffold
Source: PLoS One. 2013 Jun 21;8(6):e66067. doi: 10.1371/journal.pone.0066067 (PMC3689688; doi:10.1371/journal.pone.0066067)
Supplement: Supporting Information S1 — (docx) [file pone.0066067.s001.docx]

**Supporting Information S1.**

NP_031399.2- Homo sapiens, XP_521602.2- Pan troglodytes, XP_864622.1- Canis familiaris, NP_001095413.1-Bos taurus, NP_062632.2-Mus musculus] NP_001013173.1-Rattus norvegicus, NP_001026407.1-Gallus gallus, NP_001038251.1-Danio rerio, NP_650620.3- Drosophila melanogaster, NP_741391.2 - Caenorhabditis elegans, NP_174809.1-Arabidopsis thaliana, NP_001064062.1-Oryza sativa, XP_002572767.1 - Schistosoma mansoni, NP_001080350.1-Xenopus laevis, XP_003249714.1-Apis mellifera, EFN66969.1 - Camponotus floridanus, NP_001026407.1-Gallus gallus, XP_001649425.1-Aedes aegypti, NP_001244517.1-Macaca mulatta, XP_003218575.1-Anolis carolinensis, ZP_05038290.1-Synechococcus sp., XP_002585418.1- Plasmodium falciparum. Accession number of Shoc2 orthologues in databases (EMBL and NCBI). Xenopus laevis Shoc2 amino acid sequences and nucleotide sequences were obtained through http://genome.jgi-psf.org/Xentr4/Xentr4.home.html.
