# Supplementary figures and images for: Functional Integration of the Conserved Domains of Shoc2 Scaffold
Source: PLoS One. 2013 Jun 21;8(6):e66067. doi: 10.1371/journal.pone.0066067 (PMC3689688; doi:10.1371/journal.pone.0066067)

Supplemental Figure 1

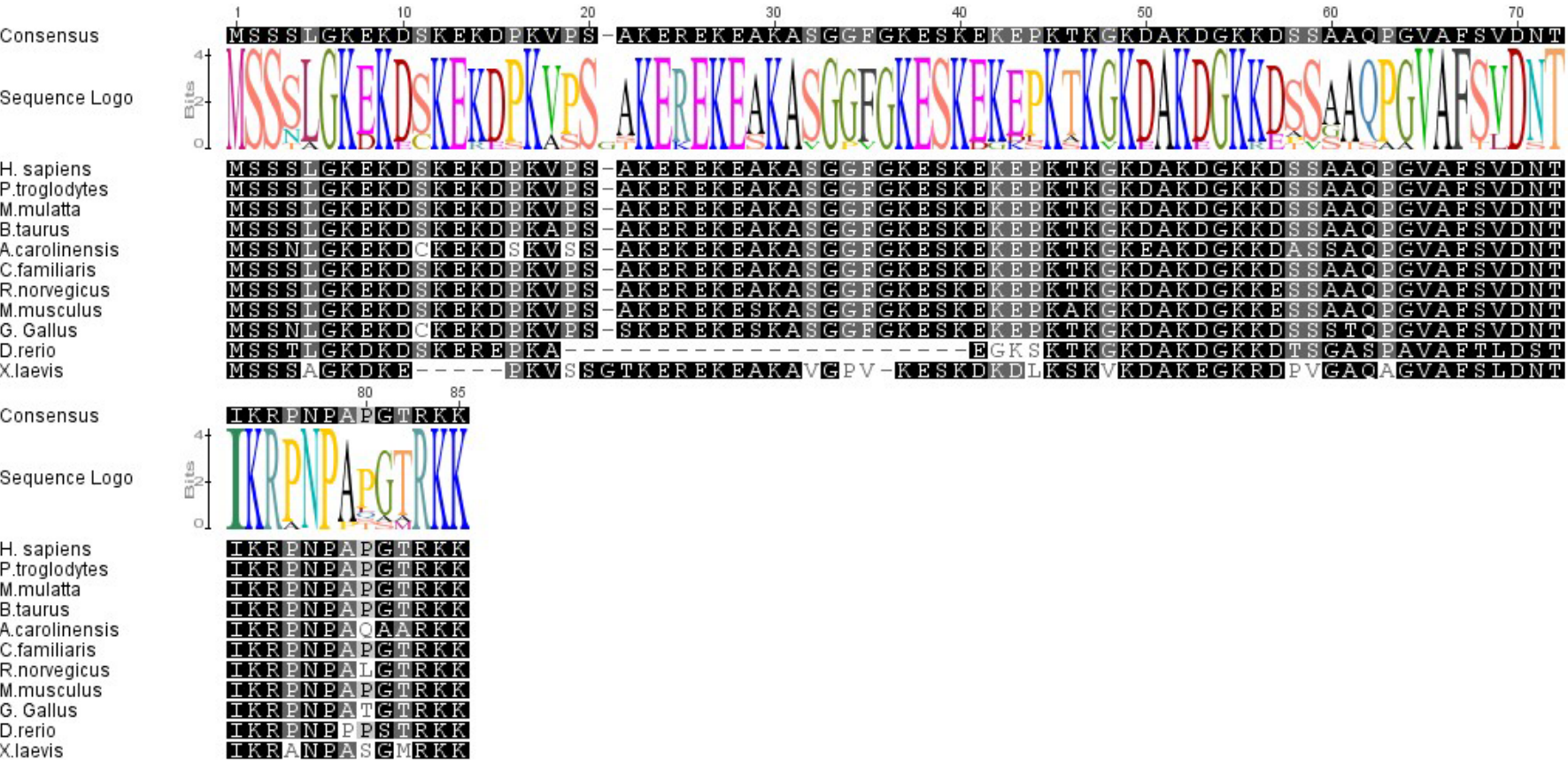

Supplement: Figure S1 — Multiple alignment of the Shoc2 N-terminal domain. An alignment of the N-terminal domains is to highlight the conservation of amino residues in vertebrate spices. The intensity of the background reflects the % of conservation of a given position within the 10 sequences (light grey>60%, dark grey>80%, black, full identity). (PDF) [file pone.0066067.s002.pdf]

## Supplementary figure 2

**A.**

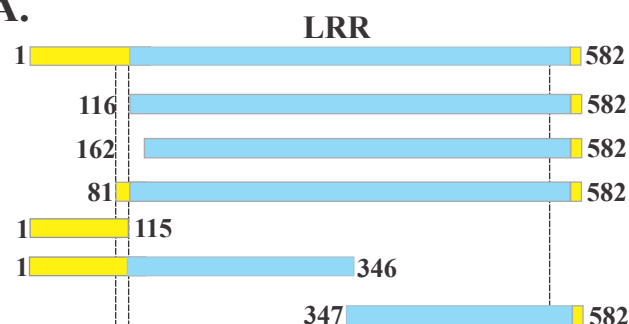

**B.**

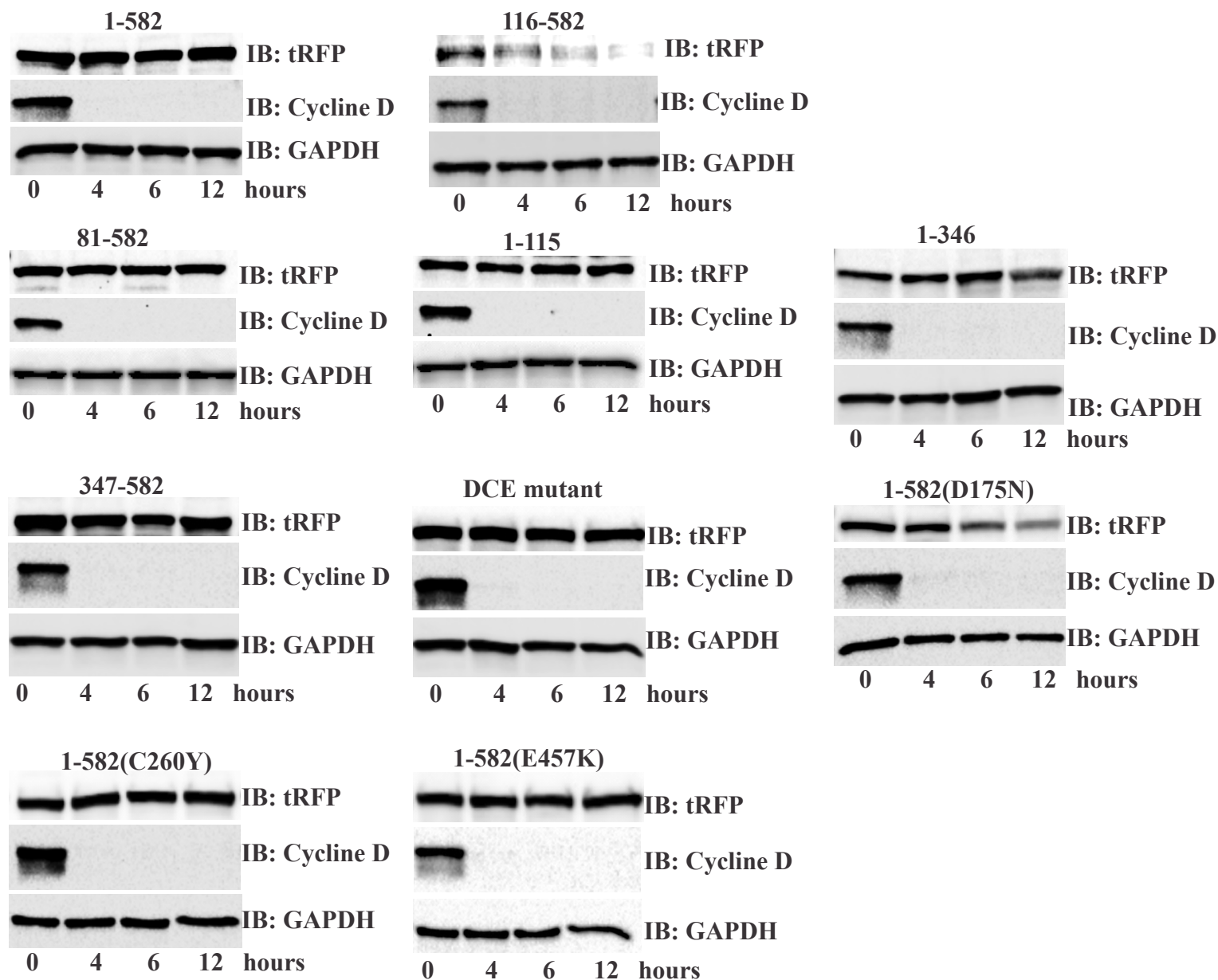

Supplement: Figure S2 — Full-length Shoc2-tRFP and Shoc2-tRFP truncated mutants have differential protein half-life. A , Schematic representation of the full-length and truncated Shoc2-tRFP constructs. B, Cos1 cells were transiently transfected with full-length Shoc2-tRFP or Shoc2-tRFP mutants. Thirty-six hours post-transfection cells were treated with 30 µM Cycloheximide for indicated times at 37°C. The lysates were probed by immuno-blotting (IB) for tRFP, Cyclin D and GAPDH (loading control). (PDF) [file pone.0066067.s003.pdf]

Supplemental Figure 3

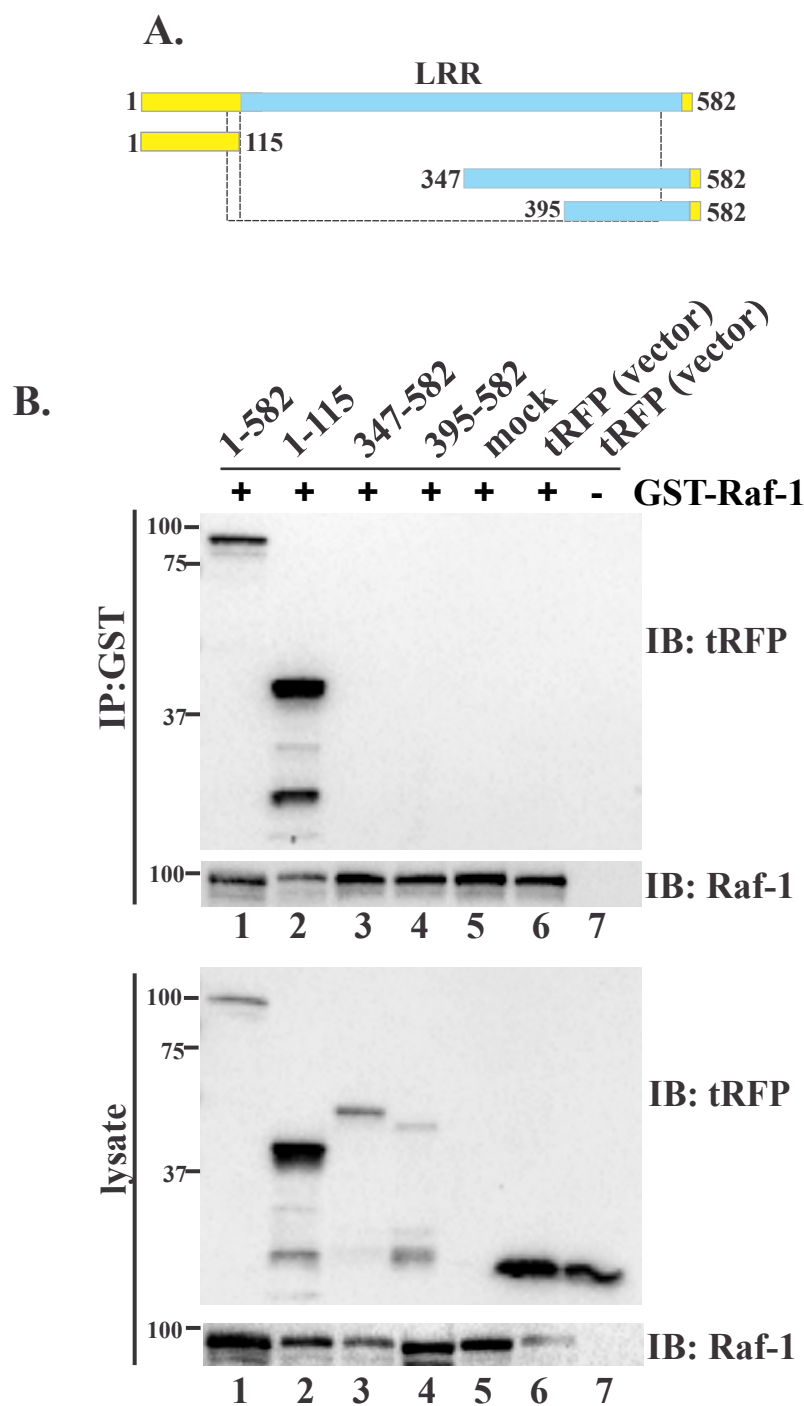

Supplement: Figure S3 — Shoc2 binding of Raf-1 in 293FT cells. A, Schematic representation of the full-length and truncated Shoc2-tRFP constructs. B, 293FT cells were transiently co-transfected with expression vectors encoding full-length or truncated tagRFP-tagged Shoc2 and GST-Raf-1. Thirty-six hours post-transfection, cells were lysed. GST-Raf-1 was precipitated with glutathione-coupled beads. The precipitated fraction was analyzed by immuno-blotting (IB) with tRFP and subsequently with Raf-1 to detect Raf-1. Cell lysates were immunoblotted with Raf-1 antibodies to monitor expression of GST-Raf-1, and tRFP antibodies to monitor expression of Shoc2 and its corresponding mutant used in panel IP. (PDF) [file pone.0066067.s004.pdf]

Supplement Figure 4.

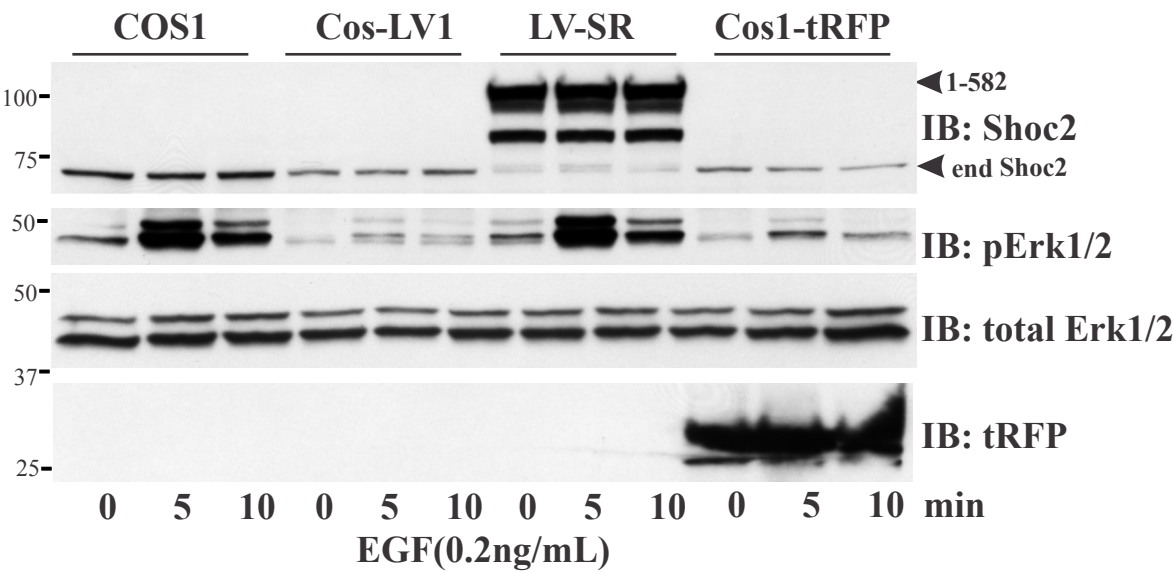

Supplement: Figure S4 — Activation of the ERK1/2 pathway in Cos1, Cos-LV1 and Cos-SR cells. Cos1, Cos-LV1, LV-SR, and Cos-1 cells transiently transfected with tRFP for 36 h were treated with EGF (0.2 ng/mL) for indicated times at 37°C. The lysates were probed by immuno-blotting (IB) for pErk1/2, Shoc2, tRFP, and total Erk1/2 (loading control). (PDF) [file pone.0066067.s005.pdf]
